# Pirfenidone Reduces Intracochlear Fibrosis Caused by Cochlear Implantation in a Guinea Pig Model

**DOI:** 10.3390/ijms27073242

**Published:** 2026-04-02

**Authors:** Kady J. Braack, Kelly L. Short, Jorjina Plester, Tylah Miles, Lee Yong Lim, Marcus D. Atlas, Jafri Kuthubutheen, Wilhelmina H. A. M. Mulders, Cecilia M. Prêle

**Affiliations:** 1 School of Human Sciences, University of Western Australia, Perth, WA 6009, Australia; kady.braack@uwa.edu.au (K.J.B.); helmy.mulders@uwa.edu.au (W.H.A.M.M.); 2 Institute for Respiratory Health, University of Western Australia, Perth, WA 6009, Australia; kelly.short@resphealth.uwa.edu.au (K.L.S.); jorjina.plester@resphealth.uwa.edu.au (J.P.); admin@resphealth.uwa.edu.au (T.M.); 3 School of Medical, Molecular and Forensic Sciences, Murdoch University, Murdoch, WA 6150, Australia; 4 Department of Pharmacy, School of Health and Clinical Sciences, University of Western Australia, Perth, WA 6009, Australia; lee.lim@uwa.edu.au; 5 Institute for Paediatric Perioperative Excellence, University of Western Australia, Perth, WA 6009, Australia; 6 Centre for Optimisation of Medicines, School of Health and Clinical Sciences, University of Western Australia, Perth, WA 6009, Australia; 7 Ear Science Institute Australia, Subiaco, WA 6008, Australia; marcus.atlas@earscience.org.au; 8 Ear Science Centre, University of Western Australia, Perth, WA 6009, Australia; 9 Medical School, University of Western Australia, Perth, WA 6009, Australia; jafri.kuthubutheen@health.wa.gov.au; 10 Department of Otolaryngology Head and Neck Surgery, Sir Charles Gairdner Hospital, Perth, WA 6009, Australia

**Keywords:** cochlear implantation, fibrosis, pirfenidone, inner ear, fibrocytes

## Abstract

While cochlear implants allow restoration of sound perception in individuals with severe to profound hearing loss, there remains significant variability in patient outcomes. A potential factor that may account for this unexplained variability is the formation of fibrosis within the cochlea after implantation. This study investigated the therapeutic potential of pirfenidone (PFD) in preventing cochlear implant-induced fibrosis and compared outcomes with dexamethasone (DEX) treated animals. The utility of PFD was determined in cultures of fibrocytes isolated from the inner ear of guinea pigs. Specifically, PFD-treatment significantly reduced p38 MAPK signalling, fibrocyte cell proliferation, migration and collagen III deposition in response to pro-fibrotic stimuli. In a guinea pig model, local hydrogel-mediated delivery of PFD to the round window at the time of implant surgery significantly reduced the amount of tissue reaction measured by micro-computed tomography at two months post-implantation (*p* = 0.0297). Specifically, a 40% decrease in implant-induced tissue reaction was observed in PFD-treated animals compared to vehicle-treated controls. Notably, no evidence of ototoxicity was observed following PFD-treatment. In contrast, a 36% decrease in the amount of tissue reaction was measured in the DEX-treated control group (*p* = 0.0436). Overall, these data demonstrate that PFD shows significant therapeutic potential in reducing cochlear implant-induced fibrosis.

## 1. Introduction

Cochlear implants allow the restoration of sound perception in individuals with severe to profound sensorineural hearing loss. Although the device was originally restricted to treatment of severe to profound hearing loss, its therapeutic scope now extends to include individuals with varying degrees of low-frequency residual hearing [1,2]. Despite the benefits of cochlear implantation in improving speech discrimination, there remains some unexplained variability in patient outcomes [3,4]. A potential factor that may explain this variability in patient outcomes is the formation of scar tissue (fibrosis) within the cochlea following implantation, which has been reported to negatively affect device function. Specifically, fibrosis has been linked to reduced battery life, reduced frequency specificity, increased electrical impedance, loss of residual hearing and in extreme cases, even device failure [4,5,6,7,8,9]. While the cellular and molecular mechanisms underlying the pathogenesis of fibrosis after cochlear implantation are not fully understood, the inflammatory response and subsequent foreign body response (FBR) are thought to drive excess extracellular matrix deposition, fibrosis and neo-ossification [10,11,12,13]. Post-mortem temporal bone studies have identified intracochlear fibrosis of varying degrees in all patients who have undergone cochlear implant surgery [14,15,16,17,18].

Several approaches aimed at reducing fibrosis following cochlear implantation have been investigated. These include changes in surgical techniques, device modifications and, more recently, pharmacological treatments [6,19,20,21]. Currently, the treatment that shows the most promise for reducing new tissue growth within the cochlea after implantation is dexamethasone (DEX), a synthetic glucocorticoid with anti-inflammatory activity [22,23]. Indeed, intracochlear DEX treatment has been shown to reduce the inflammation and immune response following implantation, ultimately resulting in reduced fibrosis and/or neo-ossification in animal models [7,21,24]. This has led to the development of a DEX-eluting electrode, a cochlear implant modified to provide sustained delivery of DEX directly into the cochlea for several months post-implantation, which has been shown to reduce electrode impedance in recent clinical trials [20,25]. Despite the reported success, DEX treatment alone is not sufficient to completely prevent fibrosis and neo-ossification [7,21]. Thus, the need for targeted, anti-fibrotic treatments suitable for use in the inner ear remains.

Previous studies have investigated other drug candidates, including anti-proliferative agents, anti-inflammatory drugs, growth factors and immune-modulators [26,27,28]. However, to the best of our knowledge, known anti-fibrotic agents have not yet been investigated for use in the inner ear. In this study, pirfenidone (PFD), a small molecule inhibitor currently approved for the treatment of idiopathic pulmonary fibrosis, was tested. PFD has been shown to effectively reduce fibrosis in a number of clinical settings: idiopathic pulmonary fibrosis, other interstitial lung diseases and myocardial fibrosis in heart failure, as well as in pre-clinical models of lung, liver, skin, kidney and cardiac fibrosis [29,30]. While the precise mechanism of action remains unknown, in the lung, PFD has been shown to reduce transforming growth factor beta 1 (TGFβ-1) signalling, fibroblast proliferation, the production of reactive oxygen species, inflammatory cell infiltration, and epithelial to mesenchymal transition [31,32,33,34]. Furthermore, previous studies have shown that local treatment of PFD reduced the foreign body response and fibrous encapsulation of implanted materials in vivo [35,36]. These characteristics identify PFD as a potential candidate for the treatment and prevention of intracochlear fibrosis following implant surgery [37].

The overarching aim of this study was to investigate the capability of PFD treatment to reduce cochlear implant-induced fibrosis in the inner ear. Firstly, in vitro studies were utilised to assess the effect and responsiveness of primary fibrocytes isolated from the inner ear to PFD. The effect of PFD on the extent and distribution of cochlear implant-induced fibrosis and neo-ossification was measured in vivo using a guinea pig model of cochlear implantation, and compared to DEX, a known and previously used treatment. PFD or DEX was applied to the round window (RW) niche using a thermosensitive hydrogel formulation at the time of implant surgery. Any ototoxic effect of drug administration was assessed in vivo using compound action potential (CAP) measurements before and after cochlear implantation surgery and drug-hydrogel administration.

## 2. Results

### 2.1. Pirfenidone (PFD) Reduces Proliferation, Collagen Deposition and the Wound Healing Response in Guinea Pig Primary Inner Ear Fibrocytes Cultures

**Figure 1:**
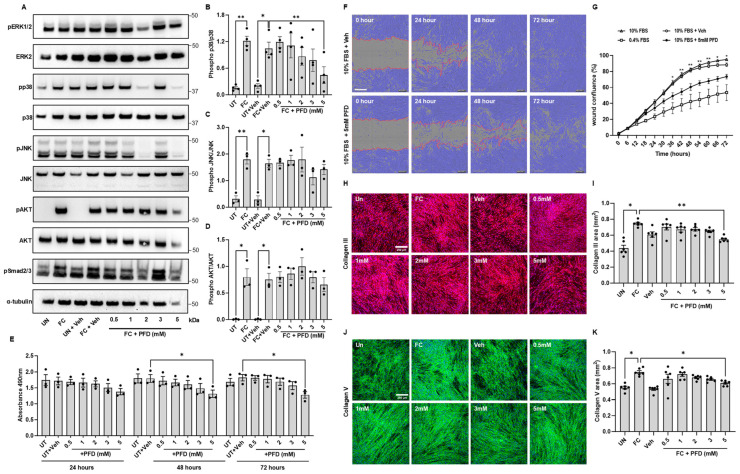
Pirfenidone (PFD) treatment altered p38 MAPK signalling, reduced proliferation, migration and collagen deposition in inner ear fibrocytes. (**A**) Western blot analysis of phosphorylated (p)ERK1/2, -P38, -JNK, -AKT and -SMAD2/3 after stimulation with a cocktail of pro-fibrotic factors (FC) or untreated (UN) and following pre-treatment with PFD or vehicle (VEH; Images are representative of *n* = 3 experiments for ERK1/2, JNK, AKT and SMAD2/3 and representative of *n* = 4 for P38). (**B**–**D**) Quantitative Western blot analysis (Data are presented as mean band intensity relative to total protein loading control, (p38 *n* = 4 experiments, JNK and AKT *n* = 3)). FC significantly increases the induction of p-p38, p-JNK and p-AKT expression at 15 min. PFD-treatment inhibits p38 pathway activation at 5 mM but has no effect on JNK or AKT pathway activation. (**E**) Treatment with 5 mM PFD resulted in a significant decrease in metabolic rate at 48 h and 72 h (*n* = 3). (**F**,**G**) The effect of PFD on wound healing, assessed using the scratch assay, demonstrated reduced wound closure at 36–72 h in fibrocytes treated with 5 mM PFD, compared to VEH control (*n* = 3 experiments with *n* = 6 replicates/experiment, scale bar 300 µm). Representative images are shown in (**F**). (**H**–**K**) Collagen III and V deposition is induced in primary inner ear fibrocyte cultures in response to treatment with a cocktail of pro-fibrotic factors (FC). PFD-treatment significantly reduced FC-induced collagen III deposition in all three experiments and collagen V deposition in two of the three experiments (*n* = 3 independent experiments with *n* > 5 replicates; data from a representative experiment is shown). Representative images are shown in H (collagen III, red) and J (Collagen V, green) (Scale bar 250 µm). Data are shown as mean ± standard error of the mean (SEM), * *p* < 0.05, ** *p* < 0.005.

Immunocytochemical staining of primary inner ear cell cultures isolated from the spiral ligament of a guinea pig cochlea confirmed the expression of caldesmon-1 and S100A10, markers previously shown to be expressed by inner ear fibrocytes [38] (Figure S1A–C). To determine the functional effect of PFD-treatment on the activation of MAPK, PI3K-AKT and SMAD signalling pathways, primary guinea pig inner ear fibrocyte cultures were treated with PFD and stimulated with a cocktail of pro-fibrotic factors (FC) or left untreated (UT). Western blot analysis was used to quantify the activation of ERK, p38, JNK, AKT and SMAD2/3 signalling pathways (Figure 1A–D). An increase in the phosphorylation of all measured pathway intermediates was observed after 15 min of FC stimulation (Figure S1D). While PFD treatment had no significant effect on FC-induced p-ERK and p-SMAD2/3 levels (*n* = 3 experiments), a decrease in p-p38 was observed in cells treated with 5 mM PFD compared to FC + VEH-treated controls (*p* = 0.0034, *n* = 4 experiments) (Figure 1A,B). Additionally, a trend towards reduced p-AKT and p-JNK levels was observed in cells cultured with 5 mM PFD; however, this was not significant (*p* = 0.0534 and 0.0914, *n* = 3 experiments) (Figure 1A,C,D). The effect of PFD-treatment on metabolic activity as a readout for cell proliferation was measured in guinea pig inner ear fibrocytes using an MTS assay. Metabolic activity was reduced in cells cultured in the presence of 5 mM PFD for 48 and 72 h (*p* = 0.0349 and 0.0142, *n* = 3 experiments, 6 replicates/experiment) (Figure 1E). To further explore the functional effects of PFD treatment on guinea pig inner ear fibrocytes, a scratch assay was performed, and wound closure was observed over 72 h. Wound closure in the scratch assay occurs as a combined result of cell survival, migration and proliferation. The wound closure rate measured by wound confluency was significantly reduced in PFD-treated cell cultures compared to VEH-treated controls from 36 to 72 h (*p* < 0.05, *n* = 3 experiments, 6 replicates/experiment) (Figure 1F,G). A key component of the excess extracellular matrix deposited around the cochlear implant is collagen. To test the ability of the guinea pig inner ear fibrocytes to deposit collagen, the macromolecular crowding (scar-in-a jar) assay was used to measure the effect of PFD on FC-induced collagen deposition. Collagen deposition was quantified using the collagen III and V isotypes, due to their role in fibrosis (Figure 1H–K, Figures S2 and S3). The average amount of collagen III and V area (mm^2^) was compared between treatment groups. FC induced a significant increase in both collagen III (*p* = 0.0108) and collagen V (*p* = 0.0189) (*n* = 3 with *n* > 5 replicates). Data from each experiment is shown (Figure 1H–K, Figures S2 and S3). PFD treatment resulted in a significant reduction in FC-induced collagen III (*p* = 0.0014) in all three experiments and collagen V (*p* = 0.0262) deposition in two of the three experiments (*n* = 3 independent experiments with *n* > 5 replicates).

### 2.2. Development and Characterisation of Drug-Loaded Hydrogels

**Figure 2:**
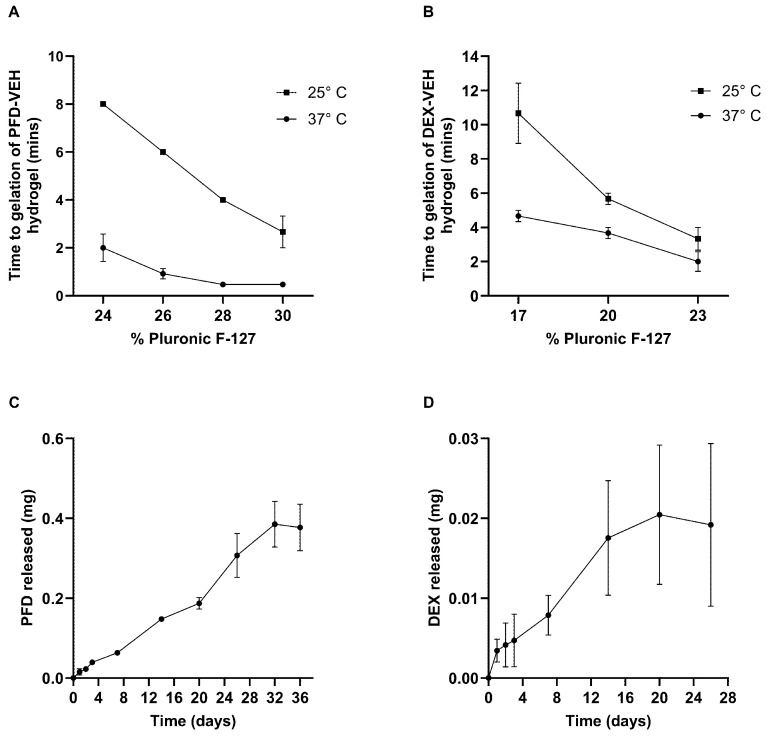
Characterisation of drug-loaded hydrogel formulations. (**A**,**B**) The gelation profile of the two vehicle-loaded hydrogel formulations for PFD and dexamethasone (DEX; VEH-PFD and VEH-DEX) was performed at 25 °C and 37 °C. (**A**) The optimum concentration of Pluronic F-127 hydrogel for PFD-mediated drug delivery was determined to be 26% with an average time to gelation of 0.93 ± 0.22 min and 6.00 ± 0.00 min at 37 °C and 25 °C, respectively. (**B**) The 17% Pluronic F-127 VEH-DEX-hydrogel formulation had an average time to gelation of 4.67 ± 0.33 min and 10.67 ± 1.76 min at 37 °C and 25 °C, respectively. (**C**) The kinetics of PFD release from the Pluronic F-127 hydrogel formulation into artificial perilymph using a modified Franz cell demonstrated sustained PFD release for 36 days, with 0.38 ± 0.06 mg released (27.1% of total drug loaded) at an estimated release rate of 10.6 μg/day. (**D**) The kinetics of DEX release from the Pluronic F-127 hydrogel formulation into artificial perilymph using a modified Franz cell. After 26 days, a total of 0.019 ± 0.010 mg of DEX was released, 17.0% of the original drug loaded, and an estimated release rate of 0.73 μg/day (*n* = 4). Data are represented as mean ± SEM, *n* = 3 experimental repeats unless otherwise stated.

The gelation characteristics of the PFD VEH hydrogel (Figure 2A) and DEX VEH hydrogels (Figure 2B) were examined. As the concentration of Pluronic F-127 in the PFD VEH hydrogel formulation increased, the time to gelation decreased. While this increase in the rate of gelation was observed at both temperatures, this trend was less pronounced at 37 °C (Figure 2A). Indeed, there was very little difference between the time to gelation at 37 °C in the hydrogels containing higher concentrations of Pluronic F-127, 28% and 30%. The optimum concentration of Pluronic F-127 to be used for further experiments was determined to be the 26% hydrogel, with an average time to gelation of 0.9 ± 0.22 and 6.0 ± 0.00 min at 37 °C and 25 °C, respectively. Overall, the DEX Pluronic F-127 hydrogel formulation showed similar characteristics, with a decrease in the time to gelation observed at the higher concentrations of Pluronic F-127. This effect was notably less pronounced at 37 °C than at 25 °C (Figure 2B). At 37 °C, the formulation containing 17% Pluronic F-127 had a time to gelation of 4.6 ± 0.33 min, and in the 23%, it was 2.0 ± 0.58 min. However, at 25 °C, the decreasing Pluronic F-127 concentration had a much larger effect, with the 17% hydrogel showing a time to gelation of 10.6 ± 1.76 min. The hydrogel formulation chosen for future experiment was the 17% Pluronic F-127, due to its use in previous studies [39,40].

Drug dissolution from the hydrogel formulations into artificial perilymph was assessed using a modified Franz cell to mimic drug release across the round window membrane and into the cochlea. PFD release from the Pluronic F-127 hydrogel followed a linear profile (Figure 2C). After 36 days, the cumulative amount of drug released was 0.38 ± 0.06 mg (*n* = 3), approximately 27.1% of the total drug loaded (1.4 mg). Consequently, the approximate release rate of PFD eluted from a 26% Pluronic F-127 hydrogel was estimated to be 10.6 μg/day. DEX release from Pluronic F-127 hydrogel also demonstrated a linear profile of drug release with a cumulative drug release of 0.019 ± 0.010 mg (*n* = 4) after 26 days, accounting for 17.0% of the 0.112 mg of DEX originally loaded (Figure 2D). The estimated release rate of DEX from 17% Pluronic F-127 hydrogel was therefore 0.73 μg/day.

### 2.3. Local PFD Treatment-Induced Weight Loss in Guinea Pigs Following Implant Surgery

**Figure 3:**
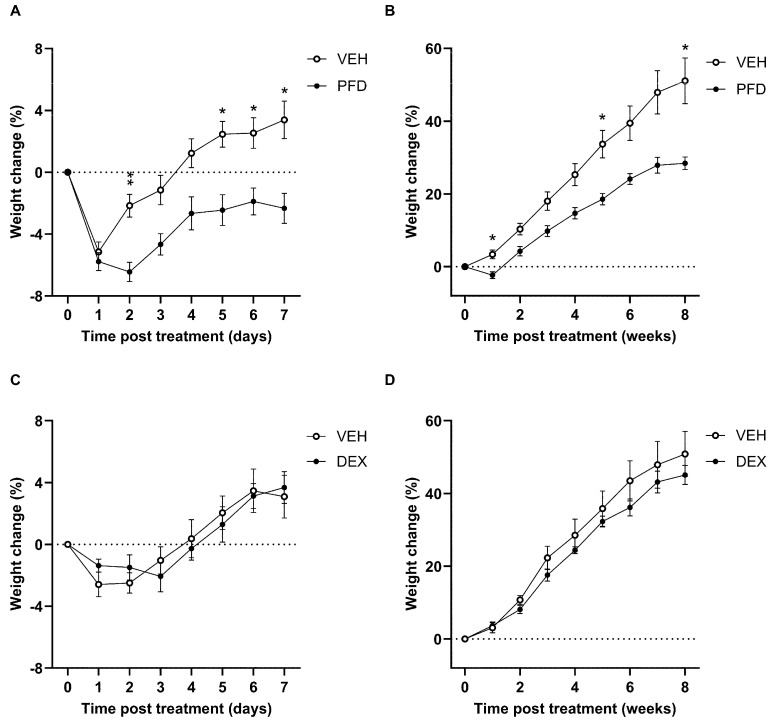
Hydrogel-mediated delivery of PFD induced weight loss in guinea pigs following cochlear implant surgery. Body weight was measured immediately prior to surgery and drug application, daily for the first 7 days post-surgery and then weekly for the duration of the experiment. Data are expressed as the % weight change for each treatment group. (**A**,**B**) Post-surgery weight loss was greater in the PFD-treated animals (*n* = 9) compared to VEH-treated controls (*n* = 13). (**A**) In the first 7 days, treatment had a significant effect on weight loss (*p* = 0.0021), with this difference reaching significance at days 2, 5, 6 and 7 (*p* = 0.0021, 0.012, 0.0253, 0.0118). (**B**) PFD treatment continued to impact body weight throughout the 8 weeks post-surgery follow-up, with PFD-treated animals gaining weight at a slower rate (*p* = 0.0068). Further, the % weight change was significantly different between PFD vs. VEH-treated animals at weeks 1, 5 and 8 (*p* = 0.0133, 0.0172, 0.035). (**C**,**D**) DEX treatment (*n* = 10) did not result in any significant differences in weight change compared to VEH-treated controls (*n* = 10). * *p* < 0.05, ** *p* < 0.005. Data are shown as mean % weight change ± SEM.

To assess any potential systemic effect of local drug delivery to the inner ear, animal body weight was monitored daily for the first 7 days following hydrogel application (Figure 3A,C), then weekly for the duration of the experiment (Figure 3B,D). A significant drop in body weight was observed in the first 7 days following PFD-treatment compared to the VEH-treated control group (Figure 3A, F (1, 20) = 12.50, *p* = 0.0021). This effect was significant at 2-, 5-, 6- and 7-day post-treatment (*p* = 0.0021, 0.012, 0.0253, 0.0118). Furthermore, the difference in % weight change measured in the PFD-treated animals remained significant throughout the following 8 weeks (Figure 3B, F (5, 38) = 4.500, *p* = 0.0068), reaching post hoc statistical significance at 1-, 5- and 8-week post treatment (*p* = 0.0133, 0.0172, 0.035). In contrast, local DEX treatment caused no significant changes in bodyweight compared to VEH controls across both the first 7 days after treatment (F (1, 144) = 0.00026, *p* = 0.987), or the following 8 weeks (F (1, 18) = 0.9206, *p* = 0.35) (Figure 3C,D). There was no significant effect of sex on weight within any treatment groups at any of the timepoints.

### 2.4. Delivery of PFD to Round Window Niche Had No Effect on Compound Action Potential (CAP) Thresholds

**Figure 4:**
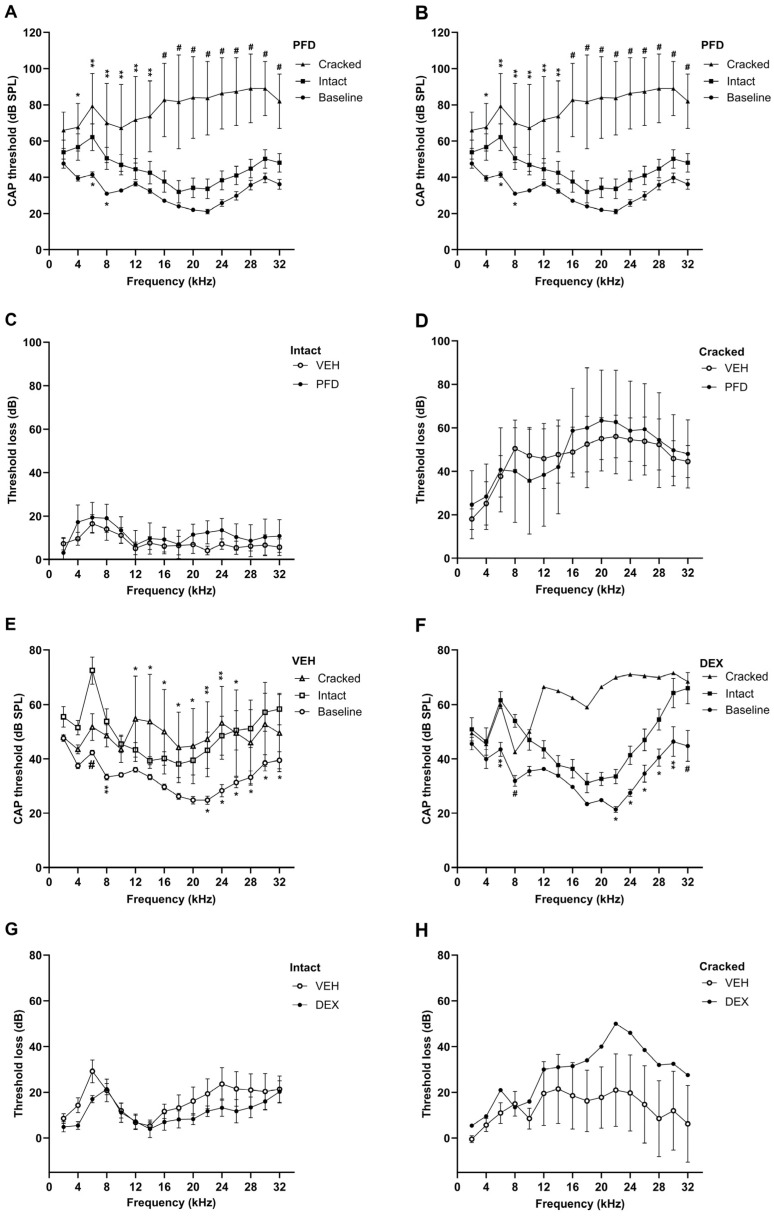
PFD treatment has no overt effect on compound action potential (CAP) thresholds. CAP thresholds (dB SPL) of the auditory nerve were measured immediately prior to (baseline) and 8 weeks following drug treatment and implantation. In the post-treatment CAP measures, treatment groups were further subdivided into animals with high levels of mechanical trauma (‘Cracked’) and those with no overt evidence of mechanical trauma (‘Intact’). (**A**) CAP thresholds of the VEH group showed that surgery had a significant overall effect (*p* < 0.0001). Animals with ‘Cracked’ cochleae (*n* = 6) had significantly higher thresholds following surgery than at baseline (*n* = 13) across all frequencies measured (2 kHz—*p* < 0.05, 4–32 kHz *p* ≤ 0.0001), while in animals from the ‘Intact’ cochleae group (*n* = 7) had significantly higher thresholds at 6 kHz (*p* = 0.0294). (**B**) Analysis of CAP thresholds in animals treated with PFD showed a similar overall effect (*p* < 0.0001), with animals in the ‘Cracked’ group (*n* = 3) had significantly higher thresholds than baseline (*n* = 9) at 4 to 32 kHz (4–14 kHz *p* = 0.0130, 0.0006, 0.0004, 0.0018, 0.0014, 0.0002; 16–32 kHz *p* < 0.0001), while animals in the ‘Intact’ group (*n* = 6) had significantly higher thresholds at 6 and 8 kHz only (*p* = 0.0226, 0.0338). (**C**,**D**) PFD treatment had no significant effect on threshold loss compared to VEH controls in either ‘Intact’ (**C**) or ‘Cracked’ (**D**) cochleae. (**E**) CAP thresholds of animals treated with DEX-VEH as compared to baseline (*n* = 10) show that both ‘Cracked’ (*n* = 4) and ‘Intact’ (*n* = 6) cochleae showed significantly increased CAP thresholds after treatment (*p* < 0.0001). This effect was significant at 6, 8, and 22 to 32 kHz for animals in the ‘Intact’ group; and at 12 to 26 kHz for animals in the ‘Cracked group.’ (**F**) CAP thresholds of animals treated with the DEX showed a significant difference after treatment as compared to baseline (*p* < 0.0001, *n* = 10). Animals with ‘Intact’ cochleae (*n* = 8) had significantly higher thresholds than baseline at 6, 8 and 22 to 32 kHz (*p* = 0.0013, <0.0001, 0.0434, 0.0175, 0.0375, 0.0164, 0.0016, 0.0001). (**G**) ‘Intact’ DEX-treated cochleae showed a significantly lower threshold loss as compared to ‘Intact’ VEH controls; however, this effect was not significant in post hoc analyses (2-way ANOVA, *p* = 0.0007). (**H**) Threshold loss of animals with ‘Cracked’ cochleae after DEX or VEH treatment; statistical analysis was not performed due to low sample size (*n* = 2). Data are represented as mean ± SEM; * *p* < 0.05, ** indicates *p* < 0.005, # indicates *p* ≤ 0.0001.

CAP thresholds were measured immediately prior to and 8 weeks following cochlear implantation with hydrogel delivery. CAP thresholds were measured within treatment groups, comparing the baseline measures with the post-treatment measures (Figure 4A,B). The presence of excessive mechanical trauma had a significant effect on CAP thresholds measured in the VEH control-treated animals (Figure 4A, *n* = 13; F (2, 368) = 307.8, *p* < 0.0001). Animals with minimal trauma (‘Intact’, *n* = 7) only had significantly higher thresholds compared to baseline at 6 kHz (*p* = 0.0294). In contrast, animals that showed high levels of mechanical trauma (‘Cracked’, *n* = 6), as a result of the cochleostomy, had significantly increased thresholds across all frequencies tested (2 kHz *p* = 0.008, 4–32 kHz *p* ≤ 0.0001). This effect of mechanical trauma was also observed with the PFD-treated group (*n* = 9, Figure 2B; F (2, 240) = 161.1, *p* < 0.0001). Once again, animals within the ‘Intact’ group (*n* = 6) had significantly higher thresholds only at 6 and 8 kHz (*p* = 0.0226, 0.0338), while animals within the ‘Cracked’ group (*n* = 3) had significantly higher thresholds from 4 to 32 kHz (4–14 kHz *p* = 0.0130, 0.0006, 0.0004, 0.0018, 0.0014, 0.0002; 16–32 kHz *p* < 0.0001). Due to the significant effect of surgical trauma within treatment groups, CAP threshold loss analysis after cochlear implantation was compared between the ‘Intact’ and ‘Cracked’ subgroups of each treatment (Figure 4C,D). PFD-treatment had no significant effect on CAP threshold loss compared to VEH in either ‘Intact’ group analysis (Figure 4C; F (15, 176) = 0.1706, *p* = 0.9998) or ‘Cracked’ group analysis (Figure 4D; F (1, 112) = 0.1216, *p* = 0.7279). Thus, PFD did not cause any additional hearing loss as compared to control animals.

Similar analyses were performed to demonstrate the effect of DEX-treatment. Surgical trauma had a significant effect on the CAP thresholds of VEH-treated animals (*n* = 10; Figure 4E; F (2, 272) = 53.98, *p* < 0.0001). Post hoc analyses demonstrate that both the ‘Intact’ (*n* = 6) and ‘Cracked’ (*n* = 4) groups had significantly worse thresholds compared to baseline; at 6, 8, and 22 to 32 kHz for ‘Intact’ animals (*p* ≤ 0.0001, 0.0055, 0.0145, 0.0064, 0.0101, 0.0172, 0.0128, 0.0119), and from 12 to 26 kHz for ‘Cracked’ animals (*p* = 0.0327, 0.0180, 0.0190, 0.0412, 0.0216, 0.0085, 0.003, 0.03). Similarly, when comparing baseline (*n* = 10) with post-implantation CAP thresholds within the DEX-treated group, surgical trauma was found to have a significant effect (Figure 4F; F (15, 272) = 4.620, *p* < 0.0001). Post hoc analyses revealed that this effect was significant at 6, 8, and 22 to 32 kHz in animals in the ‘Intact’ group (*p* = 0.0013, <0.0001, 0.0434, 0.0175, 0.0375, 0.0164, 0.0016, 0.0001, *n* = 8). The same analyses could not be performed in DEX-treated animals with ‘Cracked’ cochleae due to the small sample size (*n* = 2). DEX-treated animals within the ‘Intact’ group had significantly less overall threshold loss as compared to the VEH-treated animals (Figure 4G; F (1, 192) = 11.80, *p* = 0.0007); however, this effect did not reach significance in post hoc analyses. Again, due to low sample size, the difference between DEX-treated animals and VEH control animals was not assessed in the ‘Cracked’ subgroups (Figure 4H).

### 2.5. PFD Treatment Significantly Reduced Intracochlear Fibrosis and Neo-Ossification in a Guinea Pig Model of Implantation

**Figure 5:**
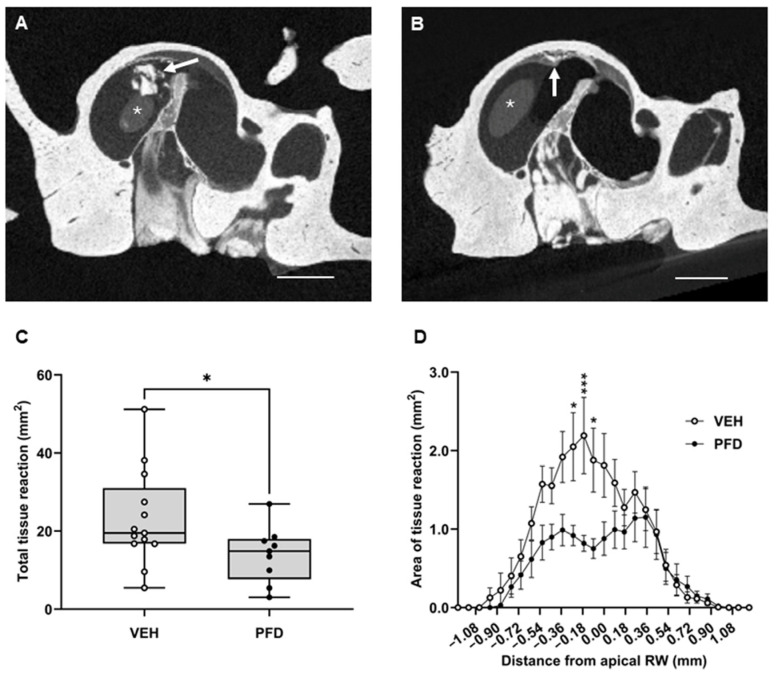
Hydrogel-mediated PFD delivery to the inner ear significantly reduces the amount of intracochlear tissue reaction measured two months post-implant surgery. VEH or PFD-loaded hydrogels were applied to the round window (RW) niche of a guinea pig cochlea at the time of cochlear implantation surgery. Intracochlear tissue reaction was quantified two months post-implant using ex vivo micro-computed tomography (μCT). (**A**,**B**) Representative images of the base of the cochlea are shown in the transaxial plane. A reduction in tissue reaction can be observed in the cochleae of PFD-treated animals (**B**) compared to VEH-treated controls (**A**); * implant, tissue reaction (white arrow), scale bar 1 mm.) (**C**) The total area of tissue reaction (mm^2^) was compared between treatments. The total amount (area) of tissue reaction measured was significantly reduced in PFD-treated animals (*n* = 9) compared to VEH-treated controls (*n* = 13, *p* < 0.0297). (**D**) The distribution of tissue reaction throughout the cochlea in reference to the apical edge of the round window (0 point) was assessed. Treatment with PFD resulted in a significant overall reduction in tissue reaction (*p* < 0.0001), which was localised to the basal areas of the round window at −0.26, −0.17 and −0.09 mm (*p* = 0.0123, 0.0006 and 0.0126). Data are represented as mean ± SEM. PFD *n* = 9, * *p* < 0.05, *** *p* < 0.0005.

The effect of PFD on the amount and distribution of the tissue reaction caused in response to implantation was determined using micro-computed tomography (μCT) as previously described [41]. Representative μCT images of osmium tetroxide-stained cochleae from PFD- and VEH-treated animals following cochlear implantation are shown (Figure 5A,B). Tissue reaction (arrows) was observed in all implanted cochleae, in close proximity to the cochleostomy site and surrounding the implant (asterisk). Qualitatively, a reduction in the amount of tissue reaction was observed in the cochleae of PFD-treated animals compared to the VEH-treated controls. Quantitative μCT analysis demonstrated a significant reduction in the overall tissue reaction in cochleae from PFD-treated animals (*n* = 9) compared to VEH-treated controls (*n* = 13). The average area of tissue reaction measured in cochleae from the PFD-treated animals was 13.99 ± 2.40 mm^2^ compared to 23.14 ± 3.40 mm^2^ in the VEH-treated group (Figure 5C). This represents a statistically significant 40% decrease in the total tissue reaction in PFD-treated animals, compared to VEH-treated controls (*p* = 0.0297). In addition to measuring the total amount of tissue reaction per cochlea, the distribution of the tissue reaction throughout the cochlea was assessed and further demonstrated a significant reduction in the measured tissue reaction in PFD-treated animals (Figure 5D; F (1, 580) = 28.39, *p* < 0.0001). In both the PFD- and VEH- treated groups, the greatest amount of tissue reaction was measured in close proximity to the RW reference point and the site of implant insertion. Notably, the PFD-induced reduction in tissue reaction was significant at −0.26 mm (*p* = 0.0123), −0.17 mm (*p* = 0.0006) and −0.09 mm (*p* = 0.0126) away from the apical edge of the RW (0 mm), demonstrating a local effect within the basal turn of the cochlea.

Extensive mechanical trauma can lead to increased fibrosis and neo-ossification. To further assess the effect of PFD-treatment on the implant-induced tissue reaction, groups were divided into ‘Cracked’ (high mechanical trauma) and ‘Intact’ (low mechanical trauma) sub-groups. The amount of tissue reaction was compared in cochleae from PFD- and VEH-treated animals in the ‘Intact’ group (Figure S4A, PFD *n* = 6, VEH *n* = 7), and then the ‘Cracked’ group (Figure S4B, PFD *n* = 3, VEH *n* = 6). PFD treatment did not have a significant effect on the total tissue reaction measured in Intact (*p* = 0.3398) or Cracked (*p* = 0.1455) compared to the respective VEH-treated controls. The difference in distribution of tissue reaction between PFD- and VEH-treated cochleae was also compared in the ‘Intact’ (Figure S4C) and ‘Cracked’ (Figure S4D) subgroups. Interestingly, PFD treatment had a significant overall effect on the distribution of tissue reaction throughout the cochlea when comparing between each subgroup, however the magnitude of this response was greater in the ‘Cracked’ group (‘Intact’ F (1, 319) = 10.60, *p* = 0.0013, Cracked F (1, 203) = 15.40, *p* = 0.0001). While analyses between treatments within ‘Intact’ cochleae did not reach post hoc significance, PFD-treatment in the ‘Cracked’ group showed significantly reduced tissue reaction at −0.17 mm away from the RW as compared to VEH control (*p* = 0.0352). Additionally, the difference in total area of tissue reaction was compared between ‘Intact’ and ‘Cracked’ cochleae within each treatment group, with no difference found within PFD- or VEH-treated cochleae (PFD *p* = 0.8806, VEH *p* = 0.2334). Interestingly, while high levels of surgical trauma had a significant effect on distribution of tissue reaction within the VEH control group (F (1, 319) = 12.43, *p* = 0.0005) which reached post hoc significance at −0.17 mm away from the apical edge of the RW (*p* = 0.0277); this same effect was not observed in PFD-treated cochleae, suggesting that PFD treatment reduced the effect of surgical trauma (F (1, 203) = 0.176, *p* = 0.675).

**Figure 6:**
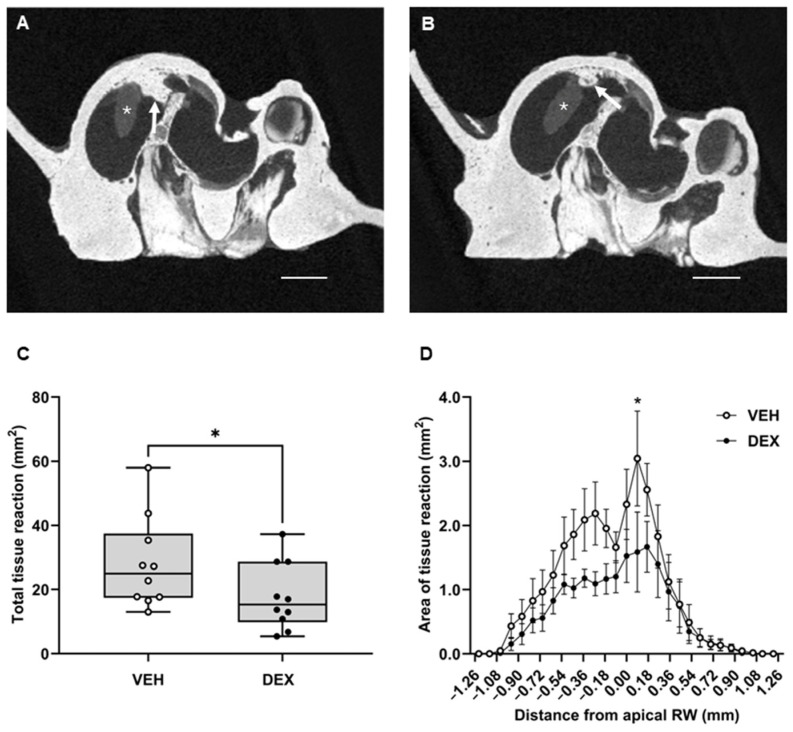
Hydrogel-mediated DEX delivery to the inner ear significantly reduces the amount of intracochlear tissue reaction measured two months post-implant surgery. VEH or DEX-loaded hydrogels were applied to the RW niche of a guinea pig cochlea at the time of cochlear implantation surgery. Intracochlear tissue reaction was quantified two months post-implant using ex vivo μCT. (**A**,**B**): Representative images of the base of the cochlea are shown in the transaxial plane. (**A**) A reduction in tissue reaction can be observed in the cochleae of DEX-treated animals (**B**) compared to VEH-treated controls (**A**); * implant, tissue reaction (white arrow), scale bar 1 mm. (**C**): The total area of tissue reaction (mm^2^) was compared between treatments. DEX-treated animals (*n* = 10) showed significantly reduced total area of tissue reaction (mm^2^) compared to VEH controls (*n* = 10, unpaired one-tailed *t*-test, *p* < 0.0436). (**D**): The distribution of tissue reaction throughout the cochlea in reference to the apical edge of the round window (0 point). Treatment with DEX resulted in a significant overall reduction in tissue reaction (*p* < 0.0001), specifically at the apical edge of the round window (0.09 mm; *p* = 0.0136). Data are represented as mean ± SEM. * *p* < 0.05.

In order to establish a treated-control to compare the effect of PFD-treatment on intracochlear tissue reaction, the effect of DEX, the most commonly tested treatment for cochlear implant-induced fibrosis, was also measured. Similar to previous studies, a significant reduction in the tissue reaction following cochlear implantation was observed qualitatively in DEX-treated animals compared to VEH-treated controls (Figure 6A,B). Quantitatively, DEX-treatment reduced the measured tissue reaction by 36% (DEX = 17.89 ± 3.30 mm^2^, VEH = 27.94 ± 4.47 mm^2^) (Figure 6C, *p* = 0.0436). Additionally, the distribution of tissue reaction in cochleae from DEX-treated animals was significantly reduced compared to VEH-treated animals (F (1, 522) = 21.40, *p* < 0.0001). Post hoc tests revealed that this effect reached significance at 0.09 mm apical from the RW (*p* = 0.0136, Figure 6D). Overall, these data suggest that DEX treatment via Pluronic F-127 hydrogel to the RW niche results in a reduced area of tissue reaction after cochlear implantation. Similarly to PFD treatment, the effect of DEX was localised to areas close to the site of insertion; however, this local effect was less pronounced than that of PFD treatment, which showed a greater reduction in tissue reaction close to the insertion site as compared to VEH controls than that of DEX-treated cochleae.

## 3. Discussion

The results of this study have demonstrated for the first time that the anti-fibrotic drug PFD can significantly reduce the amount of fibrosis and neo-ossification post cochlear implant in a guinea pig model in vivo. Initially, the responsiveness of inner ear fibrocytes to PFD was evaluated with in vitro studies showing that PFD reduced inner ear fibrocyte cell proliferation, migration, collagen deposition and altered signalling pathways induced in response to pro-fibrotic mediators. Although these anti-fibrotic effects of PFD treatment are similar to those reported in previous studies using other in vitro fibrotic disease models, such as lung, ocular and cardiac fibroblasts, there was notable variability in the response in inner ear fibrocytes [31,42,43]. For example, although PFD treatment significantly reduced p38 MAPK pathway activation in guinea pig fibrocytes isolated from the inner ear, PFD treatment had no significant effect on ERK, JNK, AKT or SMAD pathway activation. In contrast, previously published studies investigating intracellular signalling pathway changes after PFD treatment have shown decreased activation of p38, SMAD3 and AKT-mediated pathways in human lung fibroblasts; decreased SMAD3 activation in nasal polyp-derived fibroblasts; and decreased AKT, ERK1/2 and JNK activation in Tenon’s (ocular) fibroblasts [31,42,44]. Interestingly, studies using Tenon’s fibroblasts have also shown increased p38 activation after PFD treatment [42]. Thus, while the mechanism of action by which PFD treatment exerts anti-fibrotic effects in inner ear fibrocytes is not fully understood, there may be variability in the cellular response to PFD across different cell types [31,42,44]. In this instance, the variability in the effect of PFD treatment on signalling pathways may be species or cell-type-specific and more work is needed to understand the mechanisms of action in this context. The results of this study, however, demonstrate that inner ear fibrocytes can be stimulated to become pro-fibrotic as evidenced by increased collagen III and V deposition in response to exposure to a combination of pro-fibrotic factors. Furthermore, in addition to altered p38 pathway activation, these cells respond to PFD treatment by reducing proliferation, migration and collagen deposition, all of which are important cellular events that contribute to fibrogenesis.

The potential therapeutic benefit of PFD treatment in the inner ear was assessed in vivo using a guinea pig model of cochlear implantation. A hydrogel formulation was developed to achieve sustained PFD delivery into the cochlea. Hydrogels increase drug availability to the inner ear through contact with the RW membrane and can be tuned to permit sustained drug release for prolonged periods [45,46]. The capacity for sustained PFD release from the hydrogel formulation was determined in vitro, with drug release observed for up to 36 days. Prolonged drug release is vital to the treatment of cochlear implant-induced fibrosis, due to the chronicity of the FBR [13]. These data demonstrate that hydrogel-mediated delivery of PFD to the cochlea at the time of cochlear implantation surgery results in a significant reduction in the amount of intracochlear tissue reaction measured using μCT. Notably, PFD-treatment had a localised effect, with the greatest reduction in tissue reaction found in areas adjacent to the round window niche, where the drug was applied. This localised drug effect is likely representative of the concentration gradient which occurs in the cochlea after administration to the RW niche, due to the passive diffusion of the drug across the RW membrane [47,48]. The observed reduction in the total amount of tissue reaction and the localised effect of treatment was more pronounced in animals with a high level of mechanical trauma. Together, these data suggest that the drug may not have effectively penetrated the cochlear fluids and tissues, and that cochleae cracked during surgery may have had higher exposure to the drug through the cochleostomy site and cracks themselves. While analysis of these proof-of-concept studies has focused on the extent of fibrosis and neo-ossification occurring at the basal turn of the cochlea, post-implant fibrosis can be found along the length of the implant, highlighting a need for treatments that overcome the concentration gradients which occur after drug delivery to the RW niche [18,49,50].

Not only did PFD effectively reduce the tissue response to implantation, but PFD treatment appeared to be safe to use with no evidence of ototoxicity due to the drug itself. Analysis of the VEH-treated animals showed hearing loss at low frequencies. The nature of this hearing loss and the apparent low penetrance of the PFD suggest that this threshold shift at low frequencies may be due to damage or changes to the middle ear structures as a result of the hydrogel application or dampening of the basilar membrane motion due to fibrosis [51,52,53,54]. Thus, while hydrogel-mediated PFD-treatment effectively reduced intracochlear tissue reaction, the clinical application of this treatment may be limited by the current delivery method. Future studies may benefit from utilising an intracochlear delivery method which allows for sustained drug release, such as an eluting electrode, intracochlear catheter, or intracochlear drug reservoir [20,37,55], which would allow full, sustained access of the drug to the cochlea, while potentially circumventing the low-frequency hearing loss caused by hydrogel administration.

Comparative in vivo studies determined the effect of DEX-treatment and, in keeping with previous reports, showed that DEX-treatment reduces the extent of new tissue growth after cochlear implantation [7,21,56,57]. Interestingly, the reduction in the tissue response measured by μCT was less in DEX-treated animals than in those treated with PFD. This was further evidenced by the observation that PFD caused a larger and more widespread local effect closer to the RW reference point (0 mm) compared to DEX. While the two treatments cannot be directly compared due to differences in the vehicle used in each drug-hydrogel formulation, these data suggest that PFD may be a more effective alternative to DEX in preventing the formation of cochlear implant fibrosis. One possible explanation for the difference in the response is the differing mechanisms of action of the two drugs. Reportedly, PFD is thought to reduce TGFβ signalling, a pathway which has been strongly implicated in the fibrotic process in the inner ear and in numerous other tissues [12,58,59,60]. In contrast, DEX is a broad-acting glucocorticoid agonist with anti-inflammatory and immunosuppressive actions, which has been shown to reduce inflammatory cytokines and pro-collagen production as a result of intracellular activation of glucocorticoid signalling [61,62,63]. Furthermore, while the specific mechanisms by which PFD acts in the inner ear remain unclear, the data presented highlight that PFD treatment leads to a significant reduction in intracochlear fibrosis.

While these data present a convincing case for the use of PFD in the prevention of cochlear implant-induced fibrosis, several limitations need to be overcome before it may be used clinically. PFD treatment led to significant weight loss in experimental animals, which is likely due to nausea and gastrointestinal side effects. These are known side effects of PFD and are commonly reported by patients [64,65]. Additionally, alternative delivery methods could be utilised in an effort to overcome the hearing loss caused by hydrogel application and to improve drug bioavailability in the cochlea. For these reasons, future studies to support clinical translation will focus on refining the treatment further by titrating the drug to establish a minimal effect dose, reducing harmful gastrointestinal side effects and evaluating alternative delivery methods to allow for better drug availability. Overall, these data identify PFD as a potential therapeutic option for the treatment of cochlear implant-induced fibrosis.

## 4. Materials and Methods

### 4.1. Animals

The guinea pig model of cochlear implantation is a routinely used model in auditory neuroscience, which has previously been established within our laboratory [41]. Forty-Three Hartley tricolour guinea pigs (*Cavia porcellus*) were sourced from Animal Care Services at the University of Western Australia (UWA) and allowed to acclimatise in the facility for a minimum of 5 days prior to intervention. Both male and female animals were used, and care was taken to distribute either sex equally between treatment groups. All animal experiments adhered to the Code of Practice of the National Health and Medical Research Council of Australia and were approved by the Animal Ethics Committee of UWA (RA/3/100/1711 & 2022/ET000051). At the time of surgery, animals were 4 to 8 weeks old and ranged from 300 to 600 g in body weight. Animals were housed in groups of 2–4 animals of the same sex (standard pen size 175 × 55 × 35 cm), provided with standard enrichment, including fresh vegetables twice daily, and maintained on a standard 12:12 h light/dark cycle.

### 4.2. Isolation and Culture of Primary Fibrocytes from the Guinea Pig Inner Ear

One guinea pig was euthanized via intraperitoneal injection of Pentobarbitone (1 mL/kg, Lethabarb, 325 mg/mL). The cochleae were removed, the spiral ligament isolated, dissected and placed into a Geltrex^TM^ (Life Technologies, Burlington, ONT, Canada) coated culture plate. Following brief incubation at 37 °C the tissue was gently overlayed with Roswell Park Memorial Institute (RPMI 1640) media (Gibco/Thermo Fisher Scientific, Waltham, MA, USA) supplemented with 10% foetal calf serum (FCS) (Bovogen, East Keilor, NSW, Australia), 200 mM L-glutamine (Gibco/Thermo Fisher Scientific) and a combination of penicillin (10,000 U/mL) with streptomycin (10,000 μg/mL) (Gibco/Thermo Fisher Scientific) (described as ‘complete media’). Cells were incubated at 37 °C, 5% CO_2_ in a humidified incubator, and once confluent, were expanded and cryopreserved. For the experiments outlined, cells were maintained in complete media and the culture media were changed every 3 days. Primary inner ear fibrocyte cell cultures were used up to passage 20.

Primary inner ear fibrocyte cultures were characterised using immunocytochemistry. Briefly, cells were seeded onto Geltrex^TM^ (Life Technologies) coated coverslips in a 6-well plate at a density of 2 × 10^5^ cells per well and grown for 72 h. Subsequently, cells were stained for F-actin using a 488-conjugated antibody to assess cell morphology. Additionally, cells were stained with caldesmon-1 and S100A10, markers previously shown to be expressed by inner ear fibrocytes [38]. Cells were then incubated with secondary antibodies Alexa Fluor’s 564 and 647 (Invitrogen/Thermo Fisher, Brisbane, QLD, Australia) for visualisation. Cells were counterstained with 4′,6-diamidino-2-phenylindole (DAPI) (Thermo Fisher Scientific) and imaged with a Nikon A1RMP multi-photon microscope.

The functional response of inner ear fibrocytes to pro-fibrotic stimuli and the effect of treatment with PFD (or VEH) was measured. The FC was prepared as previously reported [66] and diluted in RPMI supplemented with 0.4% FCS (Bovogen), 200 mM L-glutamine (Gibco/Thermo Fisher Scientific) and a combination of penicillin (10,000 U/mL) with streptomycin (10,000 μg/mL) (Gibco/Thermo Fisher Scientific) (described as ‘0.4% FCS complete media’). The FC contained 5 ng/mL transforming growth factor-beta (TGF-β) (R&D system, Minneapolis, MN, USA), 10 ng/mL tumour necrosis factor-alpha (TNF-α) (PeproTech Inc./Thermo Fisher Scientific, Cranbury, NJ, USA), 5 µM Oleoyl-L-α-lysophosphatidic acid sodium salt (LPA) (Sigma-Aldrich, St. Louis, MO, USA) and 5 µM Platelet-derived growth factor (PDGF-AB) (PeproTech Inc./Thermo Fisher Scientific). Cells were treated with increasing concentrations of PFD drug or VEH, and responses were measured by Western blot, to investigate signalling pathway activation; MTS assay, to assess changes in cell metabolic activity as a surrogate measure of proliferation; wound healing scratch assay, a measure of migration; and macromolecular crowding assay (Scar-in-a-jar assay) was used to measure changes in collagen deposition.

### 4.3. Western Blot Analysis of Activated Signalling Pathways

Western blotting was performed as previously described [67]. Briefly, primary fibrocyte-like cells were grown to confluence and quiesced overnight prior to treatment. The cells were pretreated with PFD for 15 min, followed by stimulation with FC and PFD treatment for a further 15 min. Cells were lysed in RIPA buffer (Thermo Fisher Scientific) containing 5 mM phenylmethlsulfonyl fluoride (PMSF) (Sigma-Aldrich, MO, USA), 1× protease inhibitor (Roche, Basel, Switzerland) and 1× phosphatase inhibitor (Thermo Fisher Scientific). 10 μg of protein was resolved on Novex^TM^ Tris-Glycine 4–12% polyacrylamide gel (Invitrogen/Thermo Fisher Scientific) and transferred onto a nitrocellulose membrane (Thermo Fisher Scientific) using the iBlot transfer system (Thermo Fisher Scientific). Membranes were blocked for 1 h at room temperature in 5% skim milk in tris-buffered saline with 0.05% Tween 20 (TBS-T), then incubated overnight with primary antibodies at 4 °C (Supplementary Table S1). Membranes were washed in TBS-T and incubated with HRP-conjugated anti-rabbit secondary antibody (Cell Signalling Technology, Danvers, MA, USA) for 1 h at room temperature; blots were washed in TBS-T again and incubated in enhanced ECL (Millipore/Sigma Aldrich, St Louis, MO, USA) for 5 min. Membranes were visualised using the iBright imaging system (Invitrogen/Thermo Fisher Scientific) and analysed using FIJI imaging software (Image J2, version 2.14.011.54f) [68]. Analysis of phosphorylated protein was performed relative to total protein amount for quantification of ERK, p38, JNK and AKT, while α-tubulin was used for the quantification of phosphorylated SMAD2/3.

### 4.4. Cell Proliferation and Wound Healing Analysis

Primary inner ear fibrocytes were plated at a density of 4 × 10^4^ cells per well in a 96-well plate (Falcon^®^/Corning, Corning, NY, USA) and grown to confluency in complete media. Cells were then serum-starved overnight in 0.4% FCS complete media. The next morning, the media was changed to complete media with or without PFD. Proliferation was measured after 24, 48 and 72 h using an MTS assay (CellTiter 96 Aqueous One Solution Cell Proliferation Assay; Promega, Madison, WI, USA) and following manufacturer instructions. Briefly, 20 µL of the reagent was added to the cell media and the cells were incubated in standard conditions for 2 h before absorbance was read at 490 nm.

Primary inner ear fibrocytes were plated at a density of 2 × 10^4^ cells per well in a 96-well imagelock microplate (Sartorius, Göttingen, Germany) and grown to confluence. The cells were then serum-starved overnight in 0.4% FCS complete media. Once confluent, the cell layer was scratched using the Incucyte wound maker tool (Sartorius), creating a wound of 800 µm in width across the well. Phosphate-buffered saline (PBS) was used to wash and remove floating cellular debris; cells were then incubated in complete media with or without PFD. Wound closure was visualised and analysed using the Incucyte S3 Live-Cell Analysis System (Sartorius).

### 4.5. Macromolecular Crowding Assay for Collagen Deposition In Vitro

The Macromolecular crowding assay (Scar-in-a-jar-assay) was performed as previously described [69]. Briefly, Primary inner ear fibrocytes were seeded at 3 × 10^4^ cells per well into an optical 96-well plate (Corning) and grown to 90% confluency over 72 h. The cells were incubated in 0.4% FCS complete media overnight and then pretreated with PFD for 15 min, followed by stimulation with the FC and continual PFD treatment in crowding media for 6 days, with media being replenished after 3 days. The deposition of collagen was detected by staining for collagen III (Rockland immunochemicals Inc., Limerick, PA, USA) or collagen V (Abcam, Cambridge, UK) and visualised with Alexa Fluor 647 (Invitrogen/Thermo Fisher Scientific). The cells were counterstained with DAPI (Thermo Fisher Scientific) and imaged with a Nikon A1RMP multi-photon microscope. Quantification of collagen deposition and cell number was determined using an Image-J macro (available on request).

See Supplementary Table S1 for detailed information regarding the antibodies used for Western blot and immunocytochemistry.

### 4.6. Preparation of Drug-Loaded Hydrogels

Stock solutions of Pluronic hydrogel were prepared using the ‘cold’ method. The desired amount of Pluronic F-127 powder (Sigma-Aldrich) was weighed and dissolved in ddH_2_O to achieve a concentration of 40% (*w*/*v*). The solution was mixed at 300 rpm on ice until the powder was completely dissolved. Stock solutions were then stored at 4 °C until use, with the active drug or VEH components added immediately prior to the experiments. Both drugs were prepared by dissolving the lyophilized powder to their respective VEH components, consisting of 2% Dimethyl sulfoxide (DMSO), 30% polyethylene glycol 300 (PEG300) and 68% ddH_2_O for PFD; and 5% DMSO, 45% PEG300 and 50% ddH_2_O, for DEX; to achieve concentrations of 80 mg/mL and 3.9 mg/mL respectively. These solutions were then heated to 50 °C with agitation at 2000 rpm for 10 min, or until completely dissolved. The solutions were allowed to cool to room temperature before being combined with the Pluronic F-127 (40%) to achieve the final formulations; with the PFD hydrogel containing 28 mg/mL of drug in a 26% Pluronic F-127 solution, while the DEX hydrogel contained 2.24 mg/mL of drug in a 17% Pluronic F-127 solution.

### 4.7. Pluronic F-127 Hydrogel Gelation

To characterise the gelation kinetics of Pluronic F-127 hydrogel in the presence of each drug, VEH, the time to gelation was assessed across a range of Pluronic F-127 concentrations. Stock solution of 40% Pluronic F-127 hydrogel combined with either the PFD VEH (2% DMSO, 35% PEG300, 68% ddH_2_O) or the DEX VEH (5% DMSO, 45% PEG300, 50% ddH_2_O) at the desired concentrations. The time taken for the VEH-loaded hydrogel to undergo sol-to-gel transition was assessed by dispensing 50 μL of the gel onto parafilm and inverting the hydrogel at set time intervals. Stationary samples upon inversion indicated that the sample had undergone sol–gel transition, while movement of the sample indicated that gelation had not yet occurred. The time for the hydrogels to transition from a liquid to a gel was assessed at both room temperature (25 °C) and body temperature (37 °C) on a heat plate. The gelation rate of the Pluronic F-127 hydrogel in the presence of PFD VEH was assessed at concentrations of 24%, 26%, 28% and 30%; while in the presence of DEX VEH, concentrations of 17%, 20% and 23% were assessed. Each concentration and time was performed in triplicate, at both temperatures.

### 4.8. Measuring Drug Release from Hydrogel In Vitro

Drug release from both the PFD and DEX Pluronic F-127 hydrogel formulations was determined using a Franz diffusion cell, which had been modified to more closely resemble drug release across the round window membrane and into the cochlea [70]. Briefly, a 0.2 mL Eppendorf tube was cut in half, and the bottom half of the tube was removed. A small hole with a diameter of 2 mm was created in the centre of the lid. A dialysis membrane (Spectra/Por 7 RC Dialysis tubing, MWCO 2000) was carefully placed between the top of the tube opening and the lid. The Pluronic F-127 drug-hydrogels were prepared as previously described, and 50 μL was loaded into the Franz diffusion cell across the dialysis membrane and placed into an incubator at 37 °C to solidify the gel. This Eppendorf was then secured to the lid of a 12-well plate, wherein wells contained 1 mL of artificial perilymph solution (NaCl 125 mM, KCl 3.5 mM, NaHCO_3_ 25 mM, CaCl_2_ 1.3 mM, MgCl_2_ 1.2 mM, NaH_2_PO_4_ 0.75 mM, and dextrose 5 mM [47]). When placing the lid onto the plate, the lid of the Eppendorf, and subsequently the dialysis membrane, is in contact with the artificial perilymph. Thus, this model allows the simulation of drug release from the hydrogel, across the round window membrane, and into the cochlear fluids. To measure drug release, 50 μL samples were taken every day for the first 14 days, and then every other day up to 28 or 36 days for the DEX and PFD experiments, respectively. To replenish the volume of solution in the well after sampling, 50 μL of artificial perilymph was added to the well after each sample, with the effect on drug concentration accounted for during analysis.

### 4.9. High Performance Liquid Chromatography

Drug release in vitro was analysed using high-performance liquid chromatography (HPLC) on an Agilent Infinity II 1260 system with separation performed on a LiChrospher 100 RP-18 (250 mm × 4 mm, 5 μm). PFD was quantified at 317 nm using an isocratic mobile phase consisting of 0.2% acetic acid and acetonitrile (77:23) with a flow rate of 1 mL/min maintained at 35 °C as previously described [71]. DEX was quantified using at 240 nm using an isocratic mobile phase of methanol and water (70:30) at a flow rate of 1 mL/min at 45 °C as previously described [72].

### 4.10. Surgical Procedures, Compound Action Potential Thresholds of the Auditory Nerve and Insertion of Inactive Cochlear Implant

Animals were premedicated with 2.5 mg/kg diazepam (Ilium, diazepam 5 mg/mL) administered intraperitoneally (IP), followed by a subcutaneous (SC) injection of 0.3 mg/kg meloxicam (Ilium, Meloxicam 5 mg/mL) for analgesia. Approximately 10 min later, animals were injected IP with a solution of ketamine (Ilium Ketamine hydrochloride 100 mg/mL, 60 mg/kg) and medetomidine (Ilium, medetomidine hydrochloride, 1 mg/mL, 0.5 mg/kg) diluted in saline. Full surgical anaesthesia was indicated through the absence of both the foot withdrawal and eye blink reflexes. Once anaesthesia was achieved, the post-auricular area was shaved, and 1 mg/kg of lignocaine (Ilium, lignocaine hydrochloride, 20 mg/mL) was administered SC to the post-auricular incision site for additional analgesia. Prior to surgery, Hartmann’s solution was administered SC, at 5 mL/kg/h for approximately 5 h, to reduce the effect of dehydration on recovery.

Full surgical procedures were performed as previously described [41]. In brief, animals underwent a bullostomy procedure to expose the basal cochlea and round window niche. Hearing thresholds were assessed by measurements of the CAP of the auditory nerve, as described previously [73,74]. Immediately following CAP recordings, a small cochleostomy was created in the basal turn of the cochlea near the RW and a custom-made non-stimulating silicone electrode (MedEL, Innsbruck, Austria) was then inserted into the cochlea to a depth of approximately 5 mm, as previously described [41]. The condition of the cochlea post-cochleostomy was observed. In particular, evidence of cracks in the lateral wall of the cochlea, visualised by the surgeon, was noted. Following implant insertion, the Pluronic F-127 hydrogels were applied, either containing the active drugs, PFD (*n* = 9) or DEX (*n* = 10) or their corresponding vehicle controls (VEH-PFD, *n* = 13, VEH-DEX, *n* = 10). The hydrogel was applied to the RW using a low-waste syringe (B Braun, Melsungen, Germany) and a blunt applicator tip (24G PTFE dispensing tip, DigiKey, Thief River Falls, MN, USA). Approximately 50 μL of active drug-Pluronic F-127 hydrogel (or VEH control) was applied to the RW, with enough volume to fill the niche. Following hydrogel application, the bullostomy was sealed using Integra Collagen Matrix dressing (Integra, Life Sciences, Princeton, NJ, USA). The incision was sutured (Rivermid™ Braided cabled Nylon suture, size 3–0, Riverpoint Medical, Portland, OR, USA), and the reversal agent’s flumazenil (Anexate, Roche, 500 μ/mL, 0.1 mg/kg) and atipamezole (Ilium, 5 mg/mL, 2.5 mg/kg) were administered intraperitoneally, and the animal was allowed to recover. Animals were monitored and weighed daily for the first 7 days following surgery, and weekly for the remaining 8 weeks.

### 4.11. Terminal Surgery and Tissue Harvest

Eight weeks following cochlear implantation surgery and drug application, animals were anaesthetized using the same procedure as for the initial surgery. CAP thresholds of the auditory nerve were assessed using the same protocol described above. Immediately following the CAP measures, animals were euthanized by IP injection of sodium pentobarbitone (1 mL/kg) as Lethabarb (325 mg/mL). Animals were then intracardially perfused using physiological saline (0.9%) followed by 4% paraformaldehyde (PFA) in 0.1 M phosphate-buffered solution (PB). The tympanic bullae were dissected out, cochleae exposed, and the apex, RW and oval window were pierced to allow for manual perfusion with 4% PFA from the apex to ensure full fixation of the intracochlear tissue. During this procedure, care was taken not to disrupt the implant in situ. Samples were stored in 4% PFA in PB for a minimum of 48 h and were subsequently washed and stored in PBS.

### 4.12. Micro-Computed Tomography (μCT) Analysis

Intracochlear tissue reaction was assessed using a novel micro-computed tomography (μCT) method, which was developed and validated in an earlier study [41]. Samples were de-identified prior to scanning, and analysis was completed under blinded conditions. Cochleae were stained with osmium tetroxide (1%) for four days prior to scanning. Cochleae were placed onto a foam insert within a 5 mL tube, and scans were acquired with the Nikon XT H225 ST, using the following parameters: 65 kV beam energy, 4.00 s exposure time, 24.0 dB gain. Scans were acquired with an effective pixel size of 9 μm with 1200 projections per rotation. Background shading corrections were performed immediately prior to each scan for 1 min and 35 s. Scans were reconstructed using CT Pro 3D (version 6.14.8938.26611) and converted to TIF image files, for consequent reorientation and quantification as previously described [41]. The measures obtained to assess tissue reaction included the sum of total tissue reaction within the cochlea and the distribution of tissue reaction throughout the cochlea. Distribution of tissue reaction throughout the cochlea is presented as compressed data points, with each point representing the sum of the area of tissue reaction across 10 consecutive points (0.09 mm).

### 4.13. Statistical Analysis

Data was graphed and analysed using GraphPad Prism (version 10.1.2). Distribution of data was determined using the Shapiro–Wilk test. Data are reported as the mean ± standard error of the mean (SEM) unless otherwise stated. Differences between treatments were considered statistically significant when *p* < 0.05. Post hoc tests (such as Tukey’s test) were run only if F was significant (*p* < 0.05) and there was no variance inhomogeneity. To assess statistical significance for in vitro experiments, a paired *t*-test was used for analyses of Western blot data, a 2-way RM ANOVA with Dunnett’s multiple comparisons was used for the MTS assay, and a 2-way RM ANOVA with Tukey’s multiple comparisons for the migration and macromolecular crowding assays. To assess statistically significant differences between treatment groups in in vivo experiments, 2-way RM ANOVA was used to assess the effect of treatment on body weight and on CAP thresholds, with post hoc Sidak’s and Dunnett’s multiple comparisons, respectively. Finally, the effect of treatment on total tissue reaction was quantified under blinded conditions and assessed using unpaired one-tailed *t*-tests, while analyses comparing the distribution of tissue reaction were assessed using a 2-way RM ANOVA with Sidak’s multiple comparisons.

## Data Availability

The raw data supporting the conclusions of this article will be made available by the authors on request.

## Supplementary Materials

The following supporting information can be downloaded at: https://www.mdpi.com/article/10.3390/ijms27073242/s1.

## Abbreviations

The following abbreviations are used in this manuscript:

DEX	Dexamethasone
PFD	Pirfenidone
VEH	Vehicle
TGFβ	Transforming growth factor-β
FC	Fibrotic cocktail
UN	Untreated
MTS	3-(4,5-dimethylthiazol-2-yl)-5-(3-carboxymethoxyphenyl)-2-(4-sulfophenyl)-2H-tetra-zolium
SEM	Standard error of the mean
CAP	Compound action potential
PFA	Paraformaldehyde
PB	Phosphate buffer
PBS	Phosphate-buffered saline
μCT	Micro-computed tomography
