# Supplementary material for: Pirfenidone Reduces Intracochlear Fibrosis Caused by Cochlear Implantation in a Guinea Pig Model
Source: Int J Mol Sci. 2026 Apr 2;27(7):3242. doi: 10.3390/ijms27073242 (PMC13072998; doi:10.3390/ijms27073242)
Supplement: Supplementary file 1 [file ijms-27-03242-s001.zip › ijms-4154478-supplementary.pdf]

Supplementary Figure & Tables.

Pirfenidone reduces intracochlear fibrosis caused by cochlear implantation in a guinea pig model

Kady J Braack[1,2], Kelly L Short[2,3], Jorjina Plester[2], Tylah Miles[2], Lee Yong Lim[4,5,6], Marcus D Atlas[7], Jafri Kuthubutheen[8,9], Wilhelmina H A M Mulders[1, †], Cecilia M Prêle[2,3, †*]

Corresponding Author: Cecilia M Prêle
Email: cecilia.prele@murdoch.edu.au

This file includes:

Figure S1
Figure S2
Figure S3
Figure S4
Tables S1

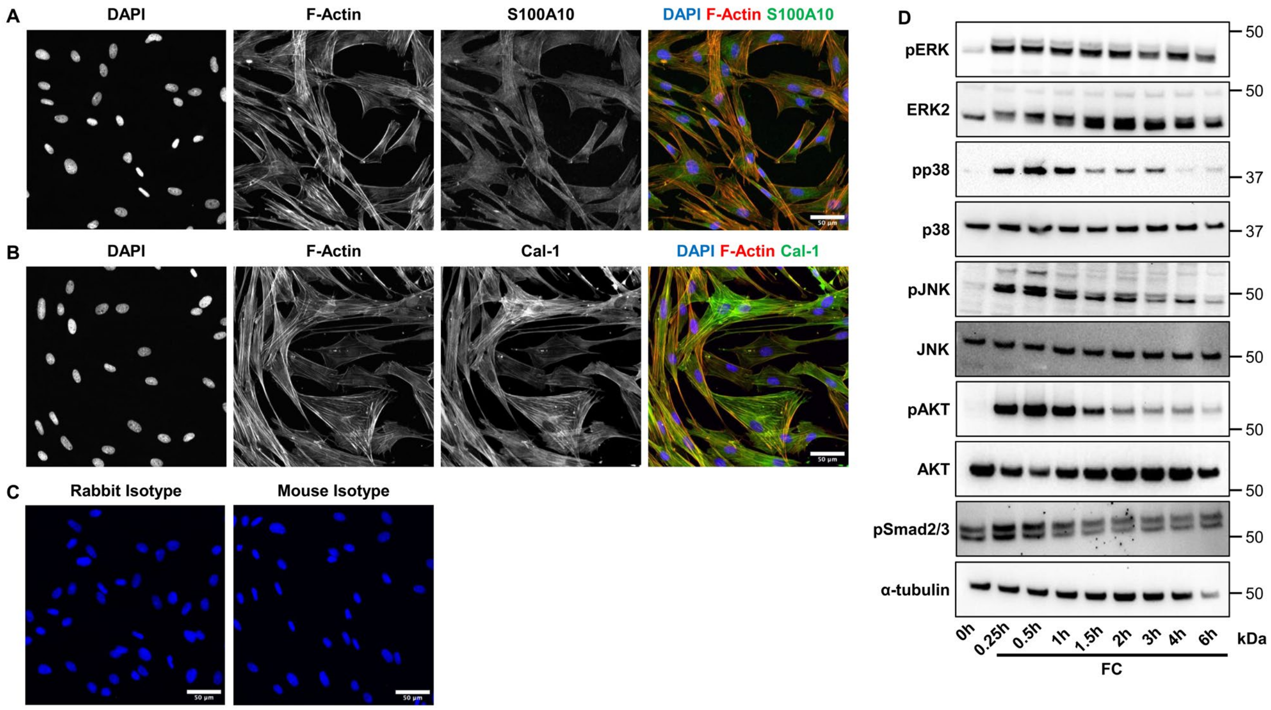

Figure S1. Characterisation of primary guinea pig inner ear fibrocytes and the induction of pro-fibrotic pathways after stimulation. A - C. Immunocytochemical characterisation of fibrocyte cell cultures obtained from the spiral ligament of a guinea pig. Cells displayed fibroblast-like morphology, shown using F-actin. Cells also expressed S100A10 (A) and Caldesmon-1 (Cal-1) (B). C. Rabbit and mouse isotypes were used as controls. Cell nuclei were visualised using DAPI. Scale bar 50μm. D. Western blot analysis of activation of ERK, p38, JNK, AKT, SMAD2/3 pathways following stimulation with a cocktail of pro-fibrotic factors (FC). The kinetics of induction were measured in protein lysates harvested from untreated cells (0h) and at 0.25, 0.5, 1, 1.5, 2, 3, 4 and 6 hours post-stimulation. These data demonstrate increased levels of phosphorylated -ERK, -p38, -JNK, -AKT and SMAD2/3 at 0.25 h (15 min) post stimulation. Total protein and α-tubulin are used as loading controls.

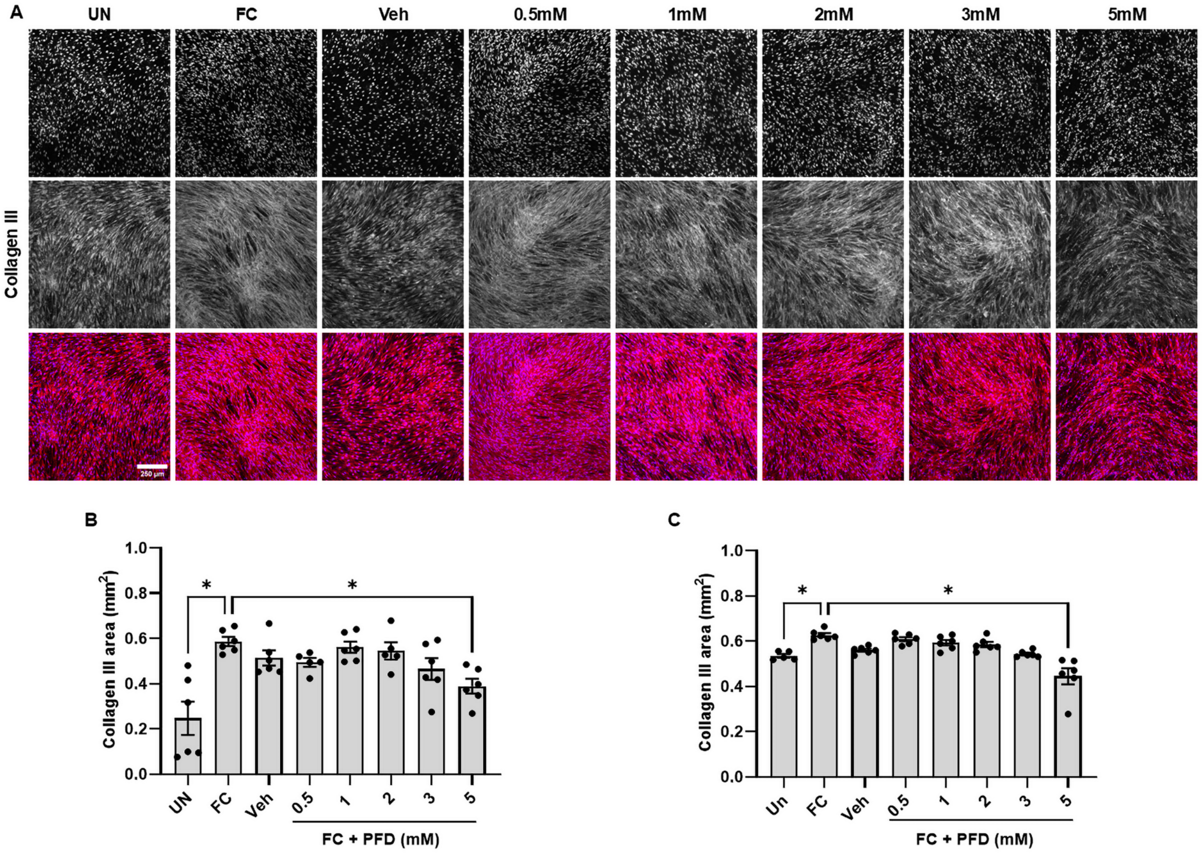

Figure S2. PFD treatment reduces collagen III deposition in primary guinea pig inner ear fibrocyte cultures. A. Collagen III deposition was measured in primary inner ear fibrocyte cultures following stimulation with a cocktail of fibrotic factors (FC). Representative images showing each of the separate channels and merged images for collagen III and DAPI staining are shown. B, C. Quantitative analysis of each of the two experimental repeats are shown. Data are represented as mean±standard error of the mean (SEM), *p<0.05, n>5 replicates per experiment).

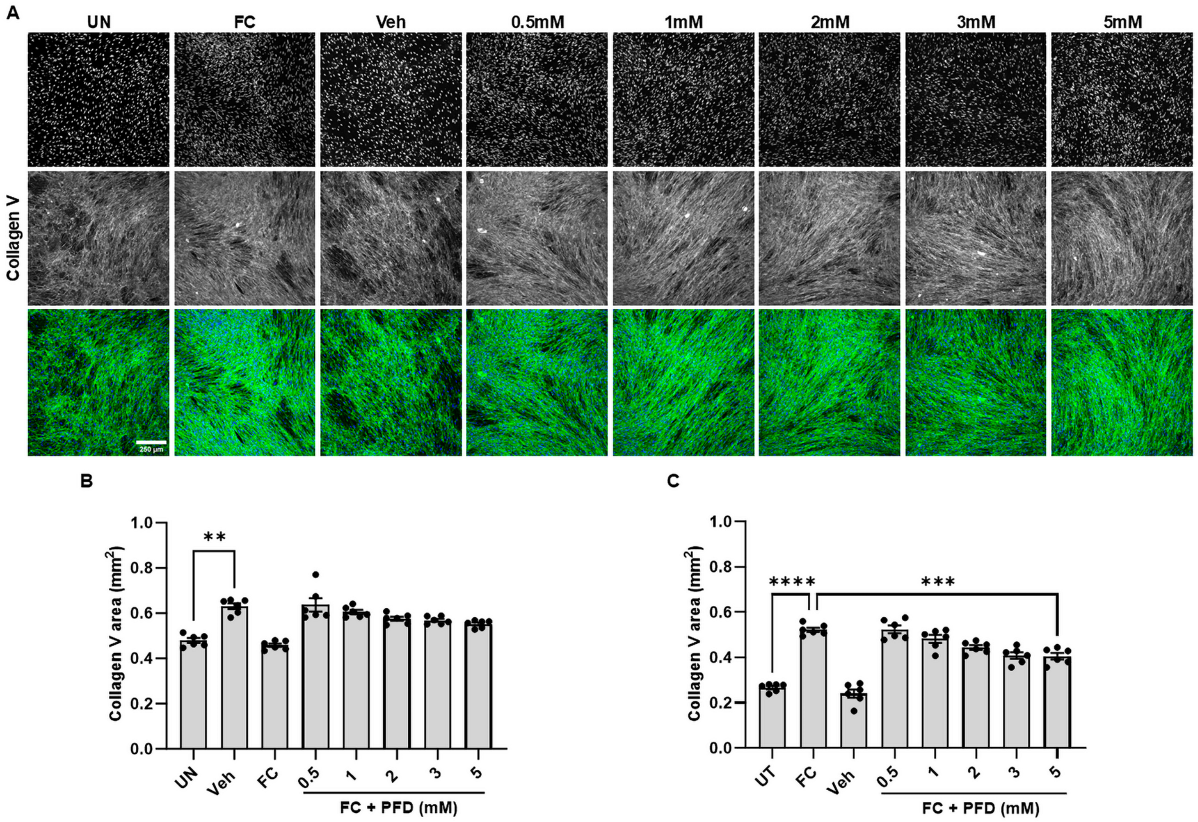

Figure S3. PFD treatment reduces collagen V deposition in primary guinea pig inner ear fibrocyte cultures. A. Collagen V deposition was measured in primary inner ear fibrocyte cultures following stimulation with a cocktail of fibrotic factors. Representative images showing each of the separate channels and merged images for collagen V and DAPI staining are shown. B, C. Quantitative analysis of each of the two experimental repeats are shown. Data are represented as mean±SEM, **p<0.005, ***p<0.0005, ****p<0.0001, n>5 replicates per experiment).

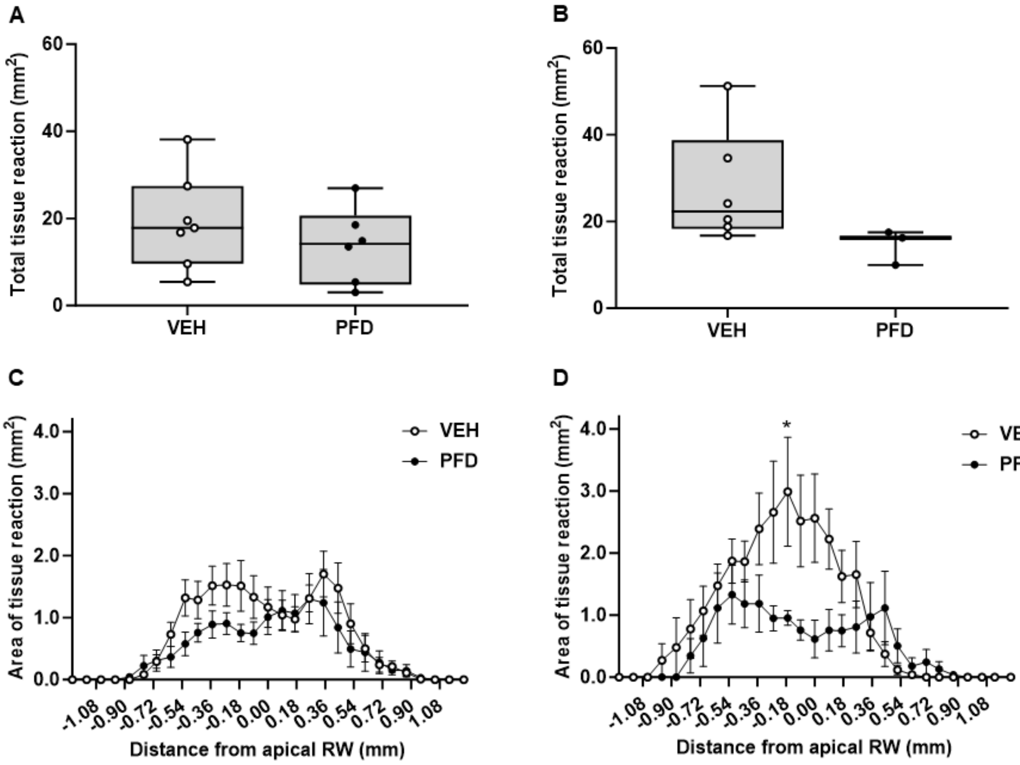

Figure S4. Effect of PFD treatment is further demonstrated in animals with high levels of mechanical trauma. Animals within each treatment group VEH or PFD were sub-divided into animals with low levels of mechanical trauma ('Intact', VEH n= 7, PFD n=6) and those with high levels of mechanical trauma ('Cracked', VEH n=6, PFD n=3). A, B. No significant difference was observed in the mean total tissue reaction measured in PFD vs VEH treated animals in either the 'Intact' sub-group (p=0.3398) or 'Cracked' cochleae group (p=0.1455). C, D. The distribution of tissue reaction throughout the basal cochlea of animals within the Intact (C) and Cracked cochleae (D) subgroups is shown. C. PFD-treatment had a significant effect on the overall distribution of tissue reaction in animals with 'Intact' cochleae (p=0.0013), however this effect was not significant in post-hoc analysis. D. In animals with 'Cracked' cochleae, PFD treatment had a significant effect on the overall distribution of tissue reaction (p=0.0001). Furthermore, post hoc analyses revealed that this effect was significant at -0.17 mm away from the RW reference point (0 mm) (p=0.0352). Data are represented as mean± SEM. *p<0.05.

Table S1. Primary antibody table.

Primary antibodies	Reference or source	Catalogue number	RRID
Phalloidin Labelling Probes - 488 (F-Actin)	Thermofisher Scientific	A12379	N/A
Caldesmon-1 rabbit monoclonal	Cell Signaling Technology	12503T	AB_2797937
S100A10 mouse monoclonal	Cell Signaling Technology	5529T	AB_10834810
Phospho-ERK1/2 rabbit monoclonal	Cell Signaling Technology	4695T	AB_390779
ERK2 rabbit polyclonal	Santa Cruz Biotechnology	SC154	AB_631459
Phospho-p38 rabbit monoclonal	Cell Signaling Technology	9215S	AB_331762
P38 rabbit polyclonal	Cell Signaling Technology	9212S	AB_330713
Phospho-SAPK/JNK rabbit polyclonal	Cell Signaling Technology	9251S	AB_331659
SAPK/JNK rabbit polyclonal	Cell Signaling Technology	9252S	AB_2250373
Phospho-AKT rabbit monoclonal	Cell Signaling Technology	4060S	AB_2315049
AKT rabbit monoclonal	Cell Signaling Technology	4691S	AB_915783
Phospho-Smad2/3 rabbit polyclonal	Cell Signaling Technology	3101S	AB_331673
Smad2/3 rabbit polyclonal	Santa Cruz Biotechnology	SC8332	AB_219319
α-Tubulin rabbit monoclonal	Cell Signaling Technology	2125S	AB_2619646
Collagen III rabbit polyclonal	Rockland Immunochemicals	600-401-105-0.1	AB_217573
Collagen V rabbit polyclonal	Abcam	ab7046	AB_305723
